# Accuracy of rapid microcapillary direct antibiotic susceptibility testing for urine samples collected with bacteriostatic boric acid from patients with suspected urinary tract infection

**DOI:** 10.1093/jacamr/dlag035

**Published:** 2026-03-31

**Authors:** Sarah Helen Needs, HoYin Lam, Jessica E Hayward, Richa Sharma, Manisha Gurung, Oliver Hancox, Julie Hart, Stephen P Kidd, Alexander Daniel Edwards

**Affiliations:** School of Pharmacy, University of Reading, Reading RG6 6UR, UK; Astratus Limited, Reading RG6 6UR, UK; Microbiology Department, Basingstoke & North Hampshire Hospital, Hampshire Hospitals NHS Foundation Trust, Basingstoke, UK; Molecular Diagnostics Hub, Royal Hampshire County Hospital, Hampshire Hospitals NHS Foundation Trust, Winchester SO22 5DG, UK; School of Pharmacy, University of Reading, Reading RG6 6UR, UK; School of Pharmacy, University of Reading, Reading RG6 6UR, UK; School of Pharmacy, University of Reading, Reading RG6 6UR, UK; School of Pharmacy, University of Reading, Reading RG6 6UR, UK; Astratus Limited, Reading RG6 6UR, UK; School of Pharmacy, University of Reading, Reading RG6 6UR, UK; Astratus Limited, Reading RG6 6UR, UK; Microbiology Department, Basingstoke & North Hampshire Hospital, Hampshire Hospitals NHS Foundation Trust, Basingstoke, UK; Molecular Diagnostics Hub, Royal Hampshire County Hospital, Hampshire Hospitals NHS Foundation Trust, Winchester SO22 5DG, UK; Astratus Limited, Reading RG6 6UR, UK; Electronics and Computer Science, University of Southampton, Southampton SO17 1BJ, UK

## Abstract

**Objectives:**

Antibiotic susceptibility testing (AST) results are needed more rapidly to support antimicrobial stewardship and improve patient outcomes. Diagnostic microbiology laboratories receive hundreds of urine samples daily from patients with suspected urinary tract infection (UTI) in both community and inpatient settings. Bacteriostatic boric acid preserves microbial contents during transport but may interfere with rapid AST methods. This study aimed to assess the accuracy of rapid microcapillary direct-from-urine (RMD) AST with suspected UTI patient urine, and to determine whether RMD AST is affected by boric acid.

**Methods:**

The overall accuracy of RMD AST was assessed with 352 diagnostic remnant urine samples collected with boric acid, for seven first-line antibiotics (ampicillin, amoxicillin/clavulanic acid, trimethoprim, nitrofurantoin, ciprofloxacin, cefalexin and cefoxitin). A further 90 urine samples were tested in duplicate with or without addition of bacteriostatic.

**Results:**

RMD AST showed a concordance with the reference method of 572/590 bacteria/antibiotic combinations (96.95%) for urine samples containing a single organism. The mean time to AST result was 5.85 h. When duplicate samples with or without boric acid were directly compared there was a categorical agreement of 158/160 (98.75%).

**Conclusions:**

The overall high accuracy of RMD AST for determining antimicrobial susceptibility to seven first-line antibiotics for UTI shows this method can deliver rapid results—without requiring additional processing—for urine samples routinely collected with boric acid from suspected UTI patients. The close agreement between duplicates with or without boric acid confirms this rapid direct method is unaffected by bacteriostatic collection.

## Introduction

Urine culture places a significant burden on routine diagnostic microbiology services due to the rising frequency of urinary tract infection (UTI).^1^ Global health research priorities for antimicrobial resistance (AMR) include improved and more rapid antibiotic susceptibility testing (AST)^2^ due to the slow and laborious nature of current culture-based phenotypic AST methods.^3^ New optical and electronic rapid detection methods could provide same-day AST results to accelerate appropriate antimicrobial prescribing.^4^ Significant emphasis has been placed on developing near-patient testing for UTI in out-of-hospital settings.^5^ The Sysmex PA-100 digital microscopy-based system provides rapid bacteriuria and AST results for five antibiotics in a near-patient setting^6^ offering potential cost benefits for uncomplicated UTI management.^7^ However, there are significant challenges to achieve widespread implementation of near-patient testing,^8^ especially for products that can only process freshly collected urine. Moreover, ∼65 million urine samples are tested annually in the UK alone,^9^ indicating a need for rapid, high-throughput, automated AST for laboratories where the most significant benefits are to be gained in complicated UTIs.^10^

An important preanalytical concern with urine culture is avoiding rapid changes in microbial contents between sample collection and laboratory testing.^11^ For over 50 years,^12^ boric acid has been added to urine as a bacteriostatic agent to reduce outgrowth of contaminants and stabilize microbiological composition,^13^ allowing sample testing up to 96 h after collection.^14^ Recent studies suggest boric acid collection is beneficial for hospitalized patients.^15^ The growing trend for pathology service consolidation means that diagnostic laboratories receive an increasing number of boric acid urine samples from both out-of-hospital and hospitalized patients.^16–18^ Current laboratory culture-based AST avoids bacteriostatic interference through the initial overnight plating step, but with direct-from-urine AST methods boric acid has the potential to interfere. We are currently unaware of any systematic study of the impact of boric acid on rapid direct-from-urine AST.

Direct-from-urine AST methods allow same-day results reporting by avoiding overnight plating, offering the potential for rapid targeted prescribing. Direct disc diffusion was first investigated in the 1970s^19^ and was more recently combined with urine cytometry and MS,^20^ or molecular methods.^21^ Separation columns,^21^ or centrifugation plus resuspension of bacteria from urine,^20^ can remove interfering substances such as boric acid. However, each additional processing step adds complexity and cost. Rapid microcapillary direct-from-urine (RMD) AST is a novel ‘dip-and-test’ technology that provides same-day accurate AST results.^22,23^ We previously reported that a single 1:10 dilution in standard culture medium was sufficient to prevent pH changes in individual urines from affecting the susceptibility results of microcapillary AST.^24^ However, the effect of bacteriostatic collection of urine samples on RMD AST remains to be systematically assessed.

The study aims were, firstly, to assess the accuracy of the RMD AST method incorporating a simple serial dilution step to avoid complex processing, for urine samples containing boric acid from suspected UTI patients. Secondly, we sought to compare duplicated urine samples with and without boric acid to examine any potential effect of bacteriostatic agent on RMD AST.

## Methods

### Ethics

Ethical approval was obtained from the NHS Health Research Authority (IRAS ID: 316558 and REC reference 22/SC/0393).

### Study design

This prospective non-interventional case–control study (ISRCTN23042419) was conducted on urine samples obtained from Hampshire Hospitals NHS Foundation Trust (HHFT). Two sample sets were investigated.

**Figure 1.**
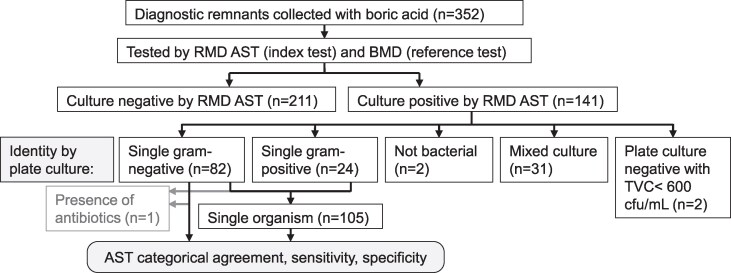
Sample flow chart detailing the diagnostic remnant samples. One single-organism Gram-negative sample was excluded based on observed growth interference consistent with presence of antibiotics in the urine.

Firstly, a total of 352 diagnostic remnant urine samples collected in boric acid red top containers were obtained from the Microbiology Laboratory at Basingstoke & North Hampshire Hospital (part of HHFT). Samples were tested in parallel by RMD AST (index test) versus in-house broth microdilution (BMD; reference test) 1–5 days after arrival at the microbiology laboratory. We cannot be certain of the concentration of boric acid in every sample, as the volume of urine in collection containers was not verified. Clinical laboratory results were obtained for these samples from HHFT, and used to confirm identity of the organisms isolated. The sample flow chart is illustrated in Figure 1.

**Figure 2.**
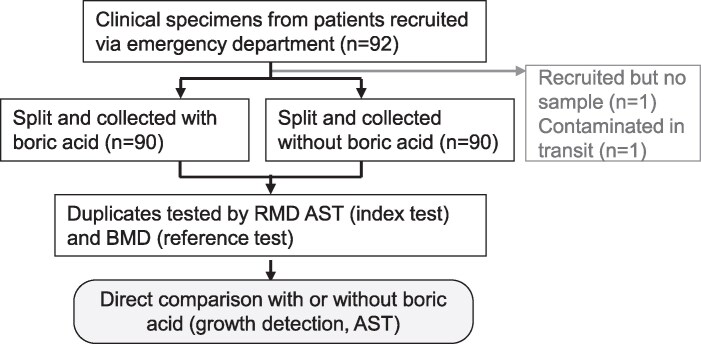
Sample flow chart detailing the samples recruited via emergency department and split into duplicates with or without boric acid.

Secondly, to directly assess the impact of boric acid on RMD AST, a further 90 fresh urine samples were collected according to hospital guidelines, using Sterilin 30 mL Universal containers without boric acid (Fisher Scientific, UK) from 92 patients consented in the Emergency Department between July and October 2024. These freshly recruited samples were split into duplicates, with 5 mL transferred into a Sterilin 30 mL Universal Boric Acid Container (Alpha Laboratories, UK) with the boric acid adjusted to achieve manufacturer-recommended concentration, and tested within 1 day of collection by index and reference methods. No clinical laboratory results were available for these 90 samples due to study protocols. The flow chart of samples is shown in Figure 2 for these Emergency Department recruited samples.

### Microbiological methods: reference BMD AST

Urine samples were streaked onto UTI ChromoSelect agar (Merck, UK) and individual colonies picked for isolation on Mueller–Hinton Agar (Merck, UK). We performed reference microplate BMD on these isolates following ISO 20776-1:2019 at the University of Reading laboratory. A standard inoculum of each isolate, adjusted by turbidity, was added to a 96-well plate containing serial antibiotic dilutions, and incubated overnight to determine MICs. Susceptibility or resistance was scored by comparing MICs with EUCAST breakpoint concentrations^25^ (Clinical Breakpoint Tables v. 13.1). Quality control reference strains including *Escherichia coli* ATCC 25922 and *Staphylococcus aureus* 29213 were tested alongside isolates. Samples were evaluated against seven first-line antibiotics for UTI: ampicillin, amoxicillin/clavulanic acid, trimethoprim, nitrofurantoin, ciprofloxacin, cefalexin and cefoxitin (Fisher Scientific, UK).

### Index method: RMD AST

Each sample was tested using an RMD AST assay cartridge containing six sets of 10 microcapillary AST test strips, as previously described.^22,23^ Briefly, microcapillary film was loaded with antibiotic solutions and cut into individual 18 mm long test strips. Batches of test strips were quality controlled with reference strains including *E. coli* ATCC 25922 and *S. aureus* 29213. Each test strip contained 10 microcapillaries: 1 growth control without antibiotic, plus 9 different conditions comprising relevant concentrations of antibiotics. Six test strips were assembled into a plastic holder to form the RMD assay cartridge. When dipped, the sample is drawn up into each microcapillary and combined with the antibiotic. Samples were diluted 1:10, 1:200 and 1:4000 in Mueller–Hinton Broth 2 (MHB; Fisher Scientific, UK) in a sterile 96-well plate to perform growth detection and AST across the wide range of bacterial cell concentrations found in urine, and to dilute the bacteriostatic agent. Resazurin (Fisher Scientific, UK) at 20 mg/L was included in this MHB dilution medium as a growth indicator. The assay cartridges are designed to be easily dipped into the serially diluted urine in the microplate. After allowing 15 s to fill the microcapillaries, end caps were applied to prevent evaporation. The assay cartridges were incubated at 35 ± 1°C within the imaging instrument, and fluorescence recorded by digital imaging at 5 min intervals.^22,23^ The longest incubation timepoint analysed was 10 h, based on our prior studies of growth detection kinetics within microcapillaries.^22^

### Bacterial identification and bacterial cell count

Urine samples were streaked onto UTI ChromoSelect agar for presumptive ID, and the identity of isolates from chromogenic growth was checked against HHFT clinical laboratory reported results. Total viable cell (TVC) counts were obtained by plating 5 µL spots of 10-fold serial dilutions of urine onto LB agar or UTI ChromoSelect agar. Colonies were counted after overnight incubation at 37°C. Bacterial growth observed on the urine-streaked plates was checked visually for evidence of growth interference; one single-organism Gram-negative sample was excluded from further analysis because of observed growth interference consistent with antibiotic residue present in the urine (Figure 1).

### Data analysis

Categorical agreement, major and very major errors, sensitivity and specificity were all calculated in accordance with ISO 20776-2:2019. Time-to-result was calculated as the time taken for detection of a positive growth signal, or the time taken to clearly discriminate susceptibility by growth inhibition from resistance where growth was not inhibited, as described previously.^26^ CIs for sensitivity and specificity were calculated using the Clopper–Pearson method. Comparisons were only possible for antibiotic/sample combinations where matched data were available for both index and reference methods. Missing data in either the index or reference methods occurred for a number of reasons. For some samples, antibiotic susceptibility could not be scored from the BMD reference method. With ciprofloxacin, samples with BMD results that fell in the area of technical uncertainty were not used in comparisons. For some individual isolates, the reference BMD method could not be completed as bacteria were not recovered after initial plating. Index method data loss arose for technical reasons when power was lost or an LED failed during incubation; this resulted in loss of image data for a set of samples after a certain timepoint. With the index method, if the bacterial growth curves obtained for a sample/antibiotic combination did not meet our quality requirements, then no susceptibility score was returned for that combination. The total number of comparisons was different for each antibiotic, and the number of comparisons for each antibiotic was reported in results tables as ‘Total samples’. When discordant results were identified between the index and reference test, the reference test was repeated.

## Results

### Accuracy of RMD AST in samples collected with boric acid

To assess the accuracy of RMD AST we first analysed all samples positive for Gram-negative bacteria, the largest pathogen population found in our study. We then analysed all single-organism samples. RMD AST was compared with the reference microplate BMD method performed by our research team on isolates taken from urine plated directly alongside the index test.

Of the 352 diagnostic remnant urine samples, the majority of culture-positive samples contained a single-organism Gram-negative bacterial species (*n* = 82); one was excluded from analysis due to the presence of an interfering substance inhibiting growth on the culture plate, presumed to be antibiotic in the urine, leaving 81 single-organism Gram-negative samples for analysis (Figure 1). Of the single-organism Gram-negative containing samples, 62 were reported by the HHFT laboratory as *E. coli*, 6 as *Proteus* spp., 11 as coliforms, 1 as *Providencia* sp. and 1 as *Klebsiella pneumoniae*. Isolates that were Enterobacteriaceae lactose fermenters but without a definitive ID from appearance on chromogenic agar were reported as coliform. Of the 24 single-organism Gram-positive samples (*n* = 24), 17 were reported by the clinical laboratory as *Enterococcus* spp., 4 as *Staphylococcus* spp. and 2 as *Streptococcus* spp., with one sample of unconfirmed identity. A further 31 samples contained two or more organisms, termed mixed culture (Figure 1).

For the diagnostic remnant samples containing a single-organism Gram-negative bacterial species, we found a concordance of 490/503 (97.42%) antibiotic/sample combinations (Table 1). We saw no obvious difference in accuracy for any individual antibiotic. The frequency of resistant organisms varied by antibiotic, as would be expected, and for antibiotics with low frequency of resistance, the lower 95% CI for sensitivity of detecting resistance was lower, reflecting the low number of samples containing a resistant organism. The presence of bacteriuria was detected with a mean time to result of 4.2 h (range 20 min to 10 h) and mean time to AST result of 5.85 h (range 2.7 to 10 h) (Table 1). In this analysis, 10 h was the latest timepoint that growth was assessed, hence the longest time to AST result was 10 h.

**Table 1.** Accuracy of direct microcapillary AST for diagnostic remnant urines containing a single Gram-negative organism compared with broth microdilution

Antibiotic	Resistant samples, *n* (%)	Total samples *n*	Sensitivity, % (95% CI)	Specificity, % (95% CI)	Categorical agreement, *n* (%)	Major error, *n* (%)	Very major error, *n* (%)	Mean time to result, h (range)
Ampicillin	41 (54.67)	75	97.56 (87.14–99.94)	97.06 (84.67–99.93)	73 (97.33)	1 (2.94)	1 (2.44)	5.85 (2.70–10.00)
Amoxicillin/clavulanic acid	23 (31.94)	72	95.65 (78.05–99.89)	93.88 (83.13–98.72)	68 (94.44)	3 (6.12)	1 (4.35)	5.91 (3.18–10.00)
Nitrofurantoin	5 (6.49)	77	100 (47.82–100)	100 (95.01–100)	77 (100)	0 (0)	0 (0)	5.89 (2.42–10.00)
Trimethoprim	25 (33.78)	74	96 (79.65–99.9)	100 (92.75–100)	73 (98.65)	0 (0)	1 (4.00)	5.81 (5.72–10.00)
Ciprofloxacin	5 (7.58)	66	100 (47.82–100)	96.72 (88.65–99.6)	64 (96.97)	2 (3.28)	0 (0)	5.86 (3.45–10.00)
Cefalexin	16 (21.92)	73	93.75 (69.77–99.84)	98.25 (90.61–99.96)	71 (97.26)	1 (1.75)	1 (6.25)	5.80 (2.78–10.00)
Cefoxitin	5 (7.58)	66	100 (47.82–100)	96.72 (88.65–99.6)	64 (96.97)	2 (3.28)	0 (0)	5.92 (3.50–10.00)
Overall	120 (23.86)	503	96.67 (91.69–99.08)	97.65 (95.59–98.92)	490 (97.42)	9 (2.35)	4 (3.33)	5.85 (2.70–10.00)

For the diagnostic remnant samples containing any single-organism Gram-negative or Gram-positive organisms we found an overall concordance of 572/590 (96.95%) antibiotic/sample combinations across the seven antibiotics for RMD AST (Table 2). We saw no obvious difference in accuracy for any of the individual antibiotics tested.

**Table 2.** Accuracy of direct microcapillary AST for diagnostic remnant urines containing a single organism (Gram-negative or Gram-positive) compared with broth microdilution

Antibiotic	Resistant samples, *n* (%)	Total samples, *n*	Sensitivity, % (95% CI)	Specificity, % (95% CI)	Categorical agreement, *n* (%)	Major error, *n* (%)	Very major error, *n* (%)
Ampicillin	43 (48.86)	88	95.35 (84.19–99.43)	97.78 (88.23–99.94)	85 (96.59)	1 (2.22)	2 (4.65)
Amoxicillin/clavulanic acid	24 (28.24)	85	91.67 (73–98.97)	95.08 (86.29–98.97)	80 (94.12)	3 (4.92)	2 (8.33)
Nitrofurantoin	5 (5.32)	94	100 (47.82–100)	100 (95.94–100)	94 (100)	0 (0)	0 (0)
Trimethoprim	31 (34.44)	90	96.77 (83.3–99.92)	96.61 (88.29–99.59)	87 (96.67)	2 (3.39)	1 (3.23)
Ciprofloxacin	10 (12.99)	77	100 (69.15–100)	97.01 (89.63–99.64)	75 (97.4)	2 (2.99)	0 (0)
Cefalexin	24 (29.27)	82	95.83 (78.88–99.89)	96.55 (88.09–99.58)	79 (96.34)	2 (3.45)	1 (4.17)
Cefoxitin	11 (14.86)	74	100 (71.51–100)	96.83 (89–99.61)	72 (97.3)	2 (3.17)	0 (0)
Overall	148 (25.08)	590	95.95 (91.39–98.5)	97.29 (95.31–98.59)	572 (96.95)	12 (2.71)	6 (4.05)

### Direct assessment of the effect of boric acid on RMD AST

To establish if media dilution is sufficient to prevent boric acid affecting microcapillary AST, we directly compared RMD AST results for split samples with or without boric acid (Table 3). We found agreement for 158/160 antibiotic/sample combinations (98.75%), clearly demonstrating that the bacteriostatic agent does not affect RMD AST performance. For the two discordant susceptibility results, growth in the presence of ciprofloxacin or cefalexin was detected without boric acid but was inhibited in the presence of boric acid.

**Table 3.** Comparison of direct urine microcapillary AST performed on duplicate samples with or without boric acid

Antibiotic	Samples,^a^ *n*	Agreement (%)
Ampicillin	21	21/21 (100)
Amoxicillin/clavulanic acid	21	21/21 (100)
Nitrofurantoin	21	21/21 (100)
Trimethoprim	22	22/22 (100)
Ciprofloxacin	25	24/25 (96)
Cefalexin	25	24/25 (96)
Cefoxitin	25	25/25 (100)
Overall	160	158/160 (98.75)

^a^Concordance was calculated for each antibiotic for all samples where susceptibility or resistance scores were reported by RMD AST in both samples, excluding combinations where susceptibility was unavailable for one sample.

### Detection of bacteriuria in samples collected with boric acid

The primary purpose of RMD AST is to report susceptibility rapidly, not to detect bacteriuria or to diagnose UTI; however, antibiotic susceptibility can only be measured if bacterial growth is detected. It was therefore vital to determine if the presence of a bacteriostatic agent affected growth detection by RMD AST in samples that were culture positive by conventional plate culture. The performance of RMD AST in detecting bacteriuria in samples containing boric acid was compared with in-house total plate counts for urine plated directly alongside RMD AST, with samples scored as positive when plate counts exceeded a 10^5^ cfu/mL threshold.^14,27,28^ A threshold of 10^5^ cfu/mL for our in-house total plate counts was chosen for this analysis based on the Kass criteria,^28^ although the diagnosis of UTI does not rely on a single cutoff cell density.^27^ Lower cell concentrations in urine can be observed in samples from UTI patients, and this threshold for the reference method could affect the calculated accuracy.^29^

When bacterial growth detection was assessed for the 352 boric acid–collected diagnostic remnant samples, the RMD AST test identified 124 positive growth samples out of 130 with in-house plate counts above 10^5^ cfu/mL (sensitivity: 95.38%; 95% CI: 90.22%–98.29%; Table 4). A total of 205 growth negatives were detected out of 222 samples with a plate count below the 10^5^ cfu/mL cutoff (specificity: 92.34%; 95% CI: 88.02%–95.48%). Three false-negative RMD AST results showed evidence of interfering substances in the urine culture plate, most likely antibiotics. The total accuracy for bacterial growth detection was 93.37% (95% CI: 90.36%–95.81%; Table 4).

**Table 4.** Comparison of bacterial growth detection by RMD AST with in-house plate counts in boric acid diagnostic remnant samples

			In-house plate counts with 10^5^ cfu/mL cutoff		
			Positive	Negative	Total
Boric acid diagnostic remnant collected samples (*n* = 352)	RMD AST	Positive	124	17	141
		Negative	6	205	211
		Total	130	222	352
			
	Sensitivity		95.38 (90.22–98.29)		
	Specificity		92.34 (88.02–95.48)		
	Accuracy		93.37 (90.36–95.81)		
	PPV		87.94 (82.18–92.02)		
	NPV		97.16 (93.98–98.68)		

PPV, positive predictive value; NPV, negative predictive value.

## Discussion

Previous studies have demonstrated that direct-from-urine AST methods can deliver accurate results.^19,30,31^ As far as we are aware, this is the first study that directly compares urine samples with or without boric acid to systematically assess the impact of bacteriostatic collection on a rapid direct-from-urine AST method. We show that rapid AST directly from urine samples stored in boric acid containers can achieve high accuracy, with an overall categorical agreement of 572/590 (96.95%) for single-organism samples stored in boric acid containers. With an average time to result of under 6 h, RMD AST can allow microbiology laboratory workflows—where boric acid collection remains essential—to rapidly report accurate AST results for UTI patients.

The analytical performance of emerging rapid AST methods and technologies has been assessed with urine samples;^3,4^ however, the presence of bacteriostatic agents is not clearly stated in many studies, especially since different laboratories have different preanalytical and collection standards. Some reports explicitly assess accuracy using samples collected with boric acid,^21^ but did not directly compare duplicates with or without bacteriostatic. Compared with direct urine disc diffusion methods,^19,30–32^ our method adds digital capture of growth kinetics, avoiding the need for manual readout. Other direct urine methods require sample processing, such as separation (e.g. centrifugation, filtration), plus adjusting bacterial cell density.^4,20^ Our method replaces these with a simple serial dilution of urine into broth, providing two benefits: the bacteriostatic agent is diluted allowing growth detection and AST; and serial dilution permits accurate AST across the high dynamic range of bacterial cell densities found in patient urine, without manual adjustment.

We assessed bacterial growth detection against an in-house plate culture using a threshold of >10^5^ cfu/mL to determine positive samples as recommended by the Kass criteria,^28^ but it should be noted that various thresholds are used for different patient and sample types.^14^ Furthermore, diagnosis of UTI is complex and should not rely on urine culture results alone. Protocols to diagnose infection in research remain heterogeneous.^29^ The purpose of RMD AST is to provide rapid susceptibility testing alongside other clinical and diagnostic factors. There was no indication that the presence of boric acid led to false negatives for growth detection that would indicate interference with growth detection by the bacteriostatic agent. Without access to further clinical information we were unable to determine if any of the samples with plate counts below the 10^5^ threshold were from patients with probable UTI. Lower plate counts are harder to interpret and can be due to colonization or contamination; and samples with lower plate counts may also have a different profile of resistance. Furthermore, these samples may be less likely to have clear comparator results from the clinical laboratory standard-of-care testing on grounds of clinical significance. For these reasons, we cannot predict how the accuracy observed for RMD AST would be affected if such samples were added to the analysis.

The time taken for bacterial growth detection to determine susceptibility varied from 2.7 to 10 h (the maximum time at which fluorescence was analysed). The variation of cell counts present in urine affects detection and AST time as urine samples with higher starting cell concentrations produce a fluorescent growth detection signal faster. We previously examined the impact of bacterial inoculum on time to growth detection and susceptibility testing,^24^ and further investigation is ongoing to determine how boric acid affects the kinetics of AST.

A strength of this study is that urine samples were taken without selection from a routine diagnostic microbiological service, representative of routine boric acid samples arriving to a centralized laboratory. However, a limitation is that the prevalence of resistant organisms for some antibiotics was low, which could lead to bias in accuracy calculations. For antibiotic/bacteria combinations where numbers of resistant organisms were low—such as nitrofurantoin—additional accuracy assessment with additional resistant isolates is now justified and further analysis of sufficient numbers of resistant organisms is planned. As this study was limited to a single site, subsequent evaluations in more sites with different resistance profiles will be required. A second limitation is that accuracy of AST was presented for 81 single-organism Gram-negative samples, which are the most common sample type, but for only 24 single-organism Gram-positive samples. However, this is as expected due to the lower prevalence of Gram-positive organisms found in human urine. Likewise, samples containing mixed organisms require more detailed and complex analysis. Current laboratory methods only record susceptibility for individual isolates. Furthermore, comparison studies would ideally test index and reference methods using the same inoculum, an approach not possible for direct testing. The microcapillary test strips used in the current study were prepared with a panel of antibiotics predominantly used for Gram-negative infections. This panel will be expanded to include antibiotics that are used more frequently with Gram-positive infections. Further studies are planned to determine the accuracy of the method with Gram-positive isolates and reference strains, and with urine samples containing a single Gram-positive organism. A detailed study of the growth of mixed organisms in microcapillary test strips is also planned.

The potential impact of fast laboratory AST on UTI management should be considered alongside the development of emerging point-of-care technologies. An initial assessment of the Sysmex PA-100 system with 278 urine samples reports broadly similar accuracy^6^ to that seen here for RMD AST, although differences in study design and population prevent a direct performance comparison. Although the Sysmex PA-100 system provides faster results, the instrument only tests a single sample at a time. In contrast RMD AST has capacity for far higher throughput, better suited to the clinical laboratory workflow. A dual strategy—combining rapid centralized laboratory direct-from-urine testing with targeted deployment of point-of-care diagnostics in remote or resource-limited settings, or for more vulnerable patients—could significantly enhance the timeliness and precision of UTI treatment.

Future research should focus on expanding the scope of validation to include Gram-positive organisms and also samples containing mixed species, which are common but remain diagnostically challenging. Additionally, studies should be adequately powered to account for varying resistance prevalence for antibiotic-pathogen combinations found across different geographies. For antibiotics with low resistance rates, such as nitrofurantoin, testing known resistant strains may be beneficial to strengthen performance assessment. Detailed assessment of how RMD AST speeds up susceptibility reporting when fully integrated within a clinical laboratory workflow, alongside detailed assessment of potential costs/benefits through health economics analysis, will likewise also be required to demonstrate the benefits of this novel rapid AST system to health systems.
